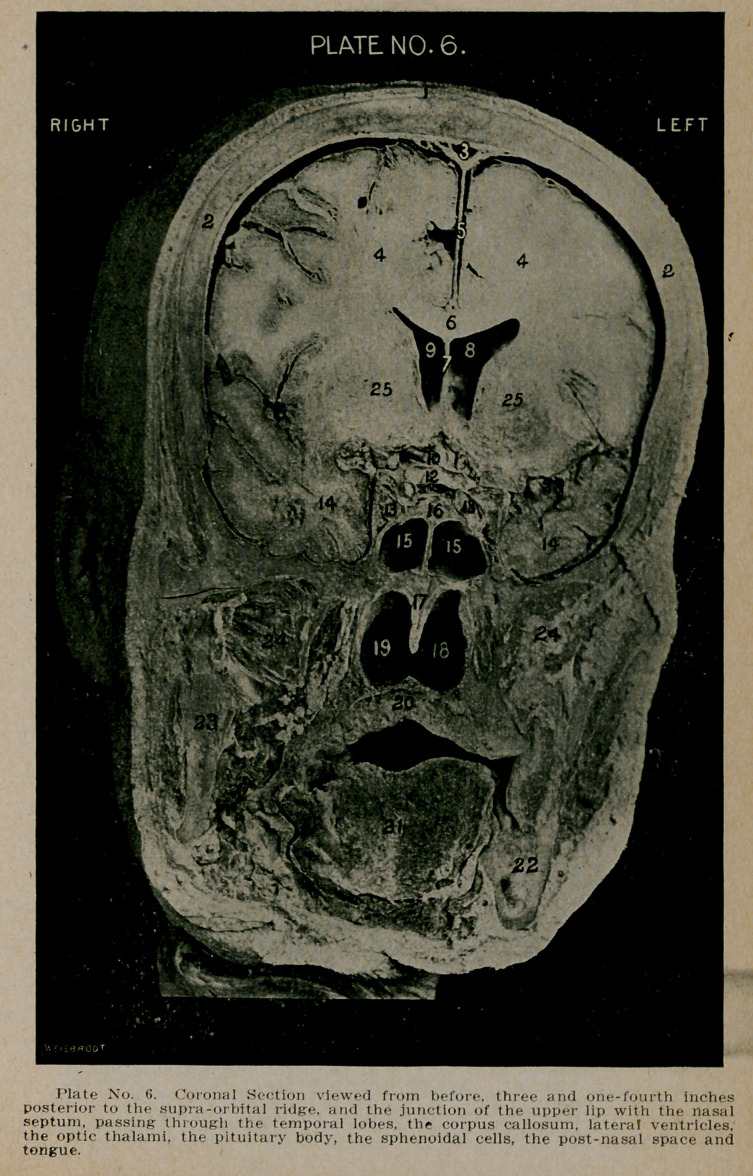# Empyema of the Nasal Accessory Sinuses

**Published:** 1915-05

**Authors:** 


**Empyema of the Nasal Accessory Sinuses.** Joseph D. Lewis of Minneapolis, St. Paul, Med. Jour. Feb., 1915, in an article on Empyema of the Nasal Accessory Sinuses, shows the accompanying plates prepared by John W. Murphy of Cincinnati, which is reproduced by courtesy of Drs. Lewis and Murphy.

Plate No. 1. Coronal Section (oblique) viewed from behind, three-fourths of an inch posterior to the supra-orbital ridge, and one inch posterior to the anterior opening of the nasal vestibule, passing through the frontal lobes, the crista galli, the frontal sinuses, the posterior portion of the right eye, and the cartilaginous portion of the septum.

### PLATE NO. 1.

1. The scalp.
2. The unusually thick tables of the frontal bones.
3. The enormously developed crista galli.
4. The anterior extremity of the frontal lobes.
5. The right and left frontal sinuses, separated by an irregular septum, deviating to the same side as the deflected nasal septum. The left sinus is much larger than the right.
6. Base of the nose, showing line of articulation with the frontal bones.
7. The nasal septum, thickened and deviating to the right side, so as to almost occlude the right nostril.
8. The enlarged left nostril.
9. The slit-like opening of the right nostril.
10. Anterior portion of the superior maxillary bone.
12. Section through the globe of the right eye, showing the sclera, ciliary muscle and the crystalline lens in position.
13. Orbital fat, surrounding the globe of the eye.
14. The Crystalline lens.

### PLATE NO. 2.

1. The scalp.
2. The very thick tables of the frontal bones.
3. The enormously developed crista galli, with the beginning of the superior longitudinal sinus, on each side of its upper extremity.
4. Section through the frontal lobes and meninges.
5. The frontal sinuses, separated by a very thick septum.
6. The base of the nose, showing line of articulation with the frontal bones.
7. The nasal septum, thickened and deviated to the right side almost occluding the right nostril.
8. The left nostril very much enlarged, by the septum deviating to the right. The turbinate bodies on the open side are very much hypertrophied, while on the closed side they are atrophied.
9. The slit-like opening of the right nostril.
10. The posterior portion of the globe of the right eye.
12. Detached retina lying in the bottom of the right eyeball.
13. Adipose tissue surrounding the globe of each eye.
14. Cornea of left eyeball.
15. The anterior or portion of the superior maxillary bone.

Plate No. 2. Coronal Section viewed from before, passing through the frontal lobes, the frontal sinuses, the posterior aspect of the right eyeball and the nasal septum.

PLATE NO. 3.

1. The scalp.
2. The tables of the frontal bones.
3. The superior longitudinal sinus.
4. Section of the cerebrum through the middle of the superior median and inferior gyri.
5. The flax cerebri, separating the two hemispheres.
6. Extension upwards and backwards of the left frontal sinus into the orbital plate.
7. Section of the olfactory bulbs resting on the cribriform plate of the ethmoid, through which filaments extend downward in the mucous membrane of the nose as far as Figure 8, which marks the lower limit of the olfactory portion of the nares.
8. The thickened and deviating septum.
9. Bulla ethmoidalis, below which (between Figures 19 and 22) the uncinate process projects into the middle meatus.
10. A large anterior ethmoidal cell or bulla ethmoidalis projecting into the middle meatus.
12. The spongy middle turbinate bodies projecting downwards from the under surface of the cribriform plate to the ethmoid. The turbinates on the right or occluded side of the nose, are much smaller than those on the left, or more open side.
13. The inferior turbinates projecting from the bony walls of the antra (Figure 18).
14. The superior maxilla, forming the roof of the mouth, and the floor of the nose.
15. The mucous surface of the upper lip.
16. The mucous surface of the lower lip.
17. The bodies of the inferior maxillary bones with the symphysis showing between them.
18. The right and left maxillary sinuses, unusually large. The floor of the right antrum is three-eights of an inch lower than the floor of the nose, while the floor of the left is one-fourth of an inch below the corresponding nasal floor.
19. The ostium maxillary, which was included in the section, on the left side. It opens into the hiatus semilunaris and thence into the middle meatus of the nose.
20. The opening of the ostium maxillare of the right antrum.
21. The upper limit of the inferior meati.
22. Middle meati. Between Figures 19 and 22, the uncinate process.
23. Section of optic nerve.
24. Section of external rectus.
25. Section of superior rectus.

Plate No. 3. Coronal Section viewed from behind, two inches posterior to the supra-orbital ridge, and one and three-quarter inches posterior to the nasal opening, passing through frontal lobes, orbital cavity, ethmoidal cells, turbinate bodies and the maxillary sinuses.

26. Section of internal rectus.
27. Section of inferior rectus.
28. Junction of vomer with superior maxilla.
29. The oral cavity.
30. Section of anterior ethmoidal cells.

### PLATE NO. 4.

1. The scalp.
2. Tables of the frontal bones.
3. The superior longitudinal sinus.
4. Section of the cerebrum through the middle of the superior, median and inferior frontal gyri.
5. The flax cerebri, separating the two hemispheres.
6. The olfactory bulbs, lying upon the cribriform plates of the ethmoid.
7. Anterior ethmoidal cells.
8. Ethmoidal bullae.
9. Hiatus semilunaris and ostium maxillare, opening into the middle fossa.
10. Maxillary sinuses. Both antra extend below the floor of the nose.
12. The middle turbinate bodies projecting from the under surface of the cribriform plate of the ethmoid.
13. The inferior turbinate bodies, projecting from the nasal wall of the maxillary sinus.
14. The thickened nasal septum.
15. The inferior nasal meatus.
16. The middle nasal meatus.
17. The superior nasal meatus.
18. The right and left nostrils.
19. The superior maxillary bone, forming the floor of the nose, and the roof of the mouth.
20. The oral cavity.
21. The cut surface of the anterior portion of the tongue.
22. The bodies of the inferior maxillary bones with the floor of the mouth between them.
23. Section of the right and left optic nerves.
24. Section of the external rectus.
25. Section of the internal rectus.
26. Section of the inferior rectus.
27. Section of the superior rectus.

Plate No. 4. Coronal Section, viewed from before, two inches posterior to the supra-orbital ridge, and one and three-quarter inches posterior to the junction of the upper lip with the nasal septum, passing through the frontal lobes, orbital, nasal and oral cavities, ethmoidal cells, spongy turbinates, maxillary sinuses, tongue and bodies of the inferior maxillary bones.

### PLATE NO. 5.

1. The scalp.
2. The skull.
3. The superior longitudinal sinus.
4. Section through the temporal lobes.
5. The flax cerebri.
6. The corpus callosum.
7. The septum lucidum, enclosing the fifth ventricle, and bounded laterally by the anterior horns of the lateral ventricles. On the left side just above Figure 13 is seen the Sylvian fissure with its contained vessels.
8. The right lateral ventricle.
9. The left lateral ventricle.
10. The optic chiasm.
12. Section of the temporal lobe. On the right side, to the left of Figure 12 is seen the cavernous sinus, containing the third, fourth, the ophthalmic division of the fifth and sixth nerves, together with the internal carotid artery.
13. The uncinate gyrus of the temporal lobe. Above Figures 12 and 13 the section passes through the foot of the anterior central (Rolandic) gyri, and the caudal extremities of the three frontal gyri.
14. A small accessory sphenoidal cell with its separate opening communicating directly with the ethmoid in front.
15. The right and left sphenoidal cavities.
16. Posterior end of vomer, or septum.
17. The choanae, or pharyngeal openings of the nostrils.
18. The posterior portion of the left middle turbinate.
19. The posterior portion of the left inferior turbinate.
20. The superior maxillary bone forming the floor of the nose and the roof of the mouth.
21. Section of the middle of the tongue.
22. Section of the inferior maxilla.
23. Section of the ascending ramus of the inferior maxillary bone.
24. Section of the superior maxillary bone.

25 and 26. Section of the temporo-maxillary muscles.

27. Section of the optic thalami.

### PLATE NO. 6.

1. The scalp.
2. Section of the skull.
3. The superior longitudinal sinus.
4. Section through the temporal lobes.
5. The flax cerebri.
6. The corpus callosum.
7. Septum lucidum, enclosing the fifth ventricle and bounded laterally by the anterior horns of the lateral ventricles.
8. The left lateral ventricle.
9. The right lateral ventricle.
10. The basilar artery.
12. The pituitary body.
13. The internal carotid artery and cavernous sinus.
14. The uncinate gyrus of the temporal lobe. Immediately above Figure 14, on the left side, is seen the cavernous sinus with its contained vessels and nerves.
15. The right and left sphenoidal cells.
16. The sella turcica.
17. The junction of the bony septum with the body of the sphenoid.
18. The post-nasal opening. On the outer wall of this space is seen the trumpet-like opening of the left eustachian tube.
19. The posterior wall of the post-nasal space. On its lateral wall is seen the right eustachian tube.
20. The superior maxillary bone forming the floor of the nose and the roof of the mouth.
21. Section of the tongue near its base.
22. The inferior maxillary bone at angle of the jaw.
23. Section of the ascending ramus of the inferior maxillary bone.
24. Section of the temporal muscles.

## Figures and Tables

**Plate No. 1. f1:**
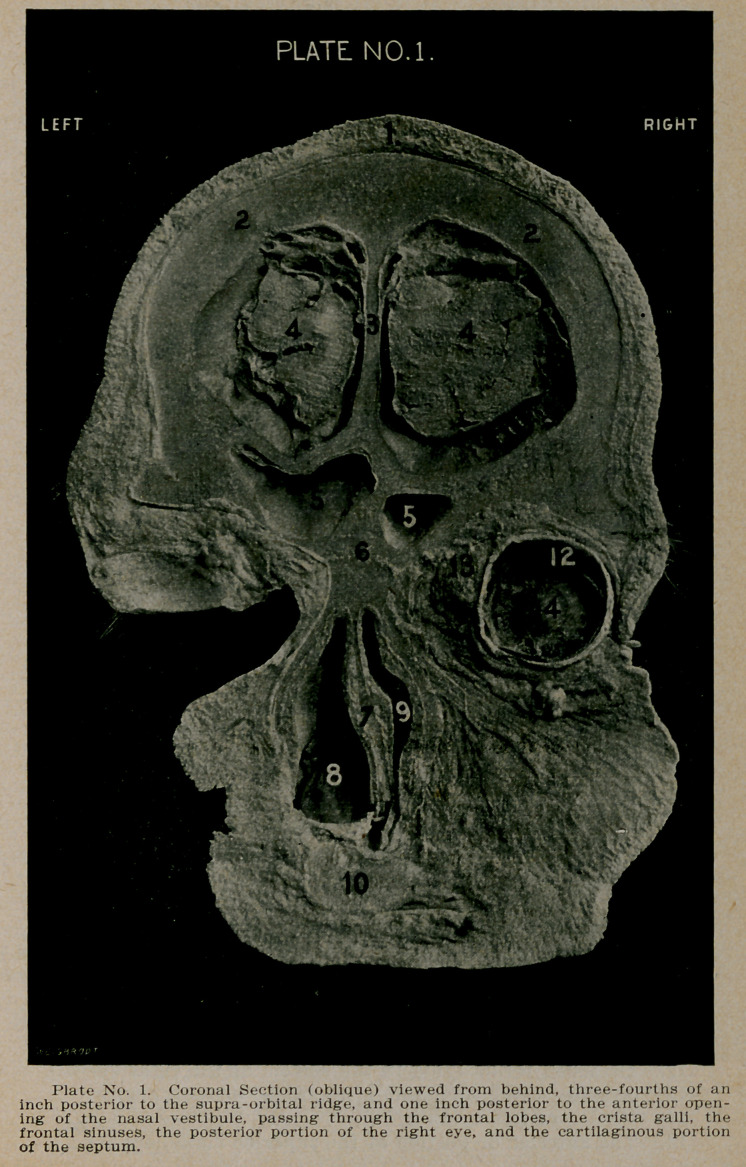


**Plate No. 2. f2:**
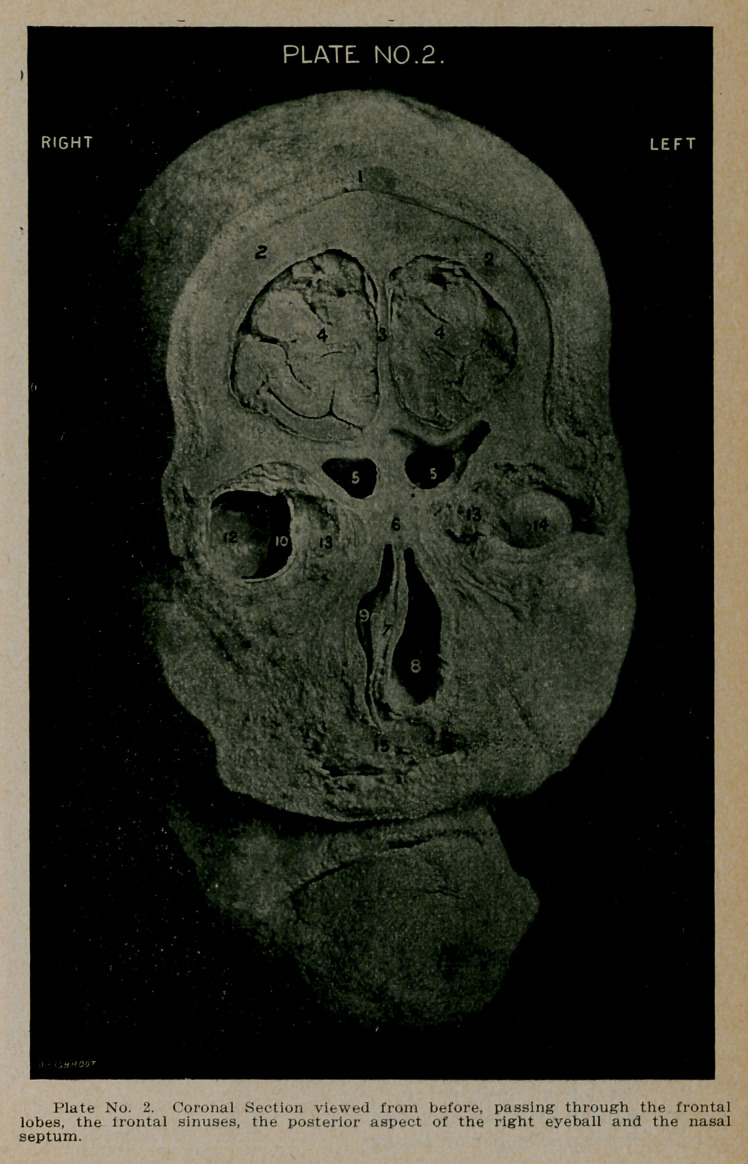


**Plate No. 3. f3:**
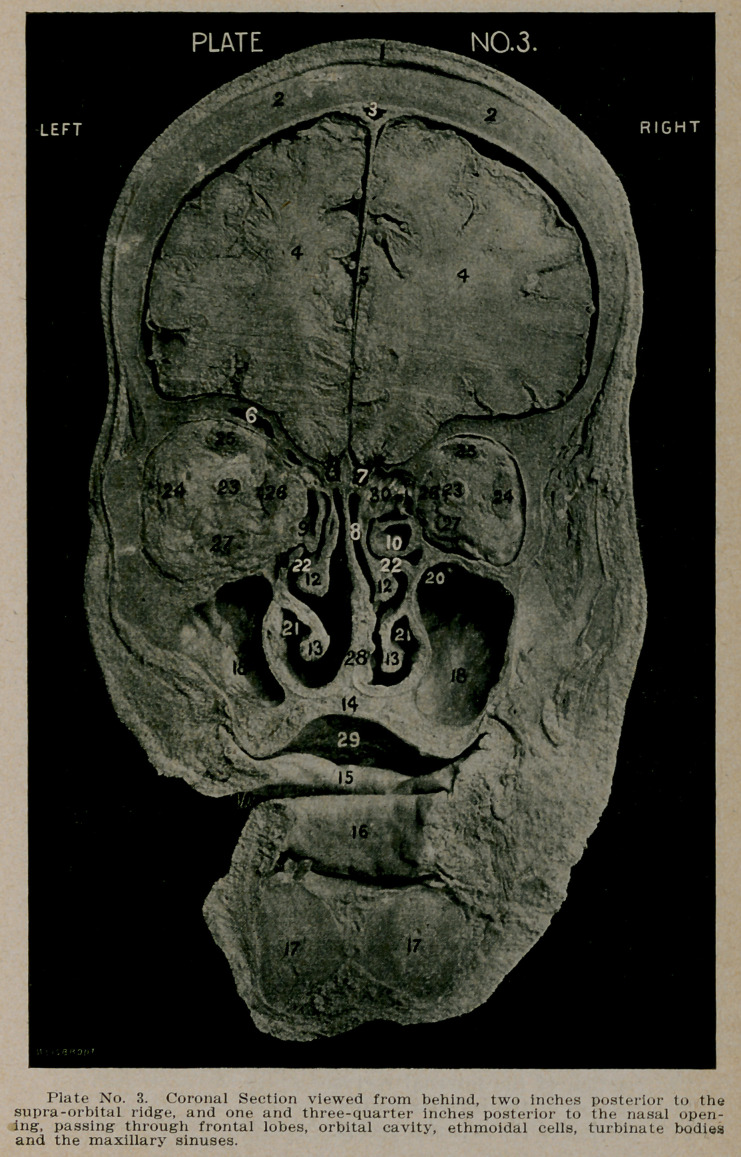


**Plate No. 4. f4:**
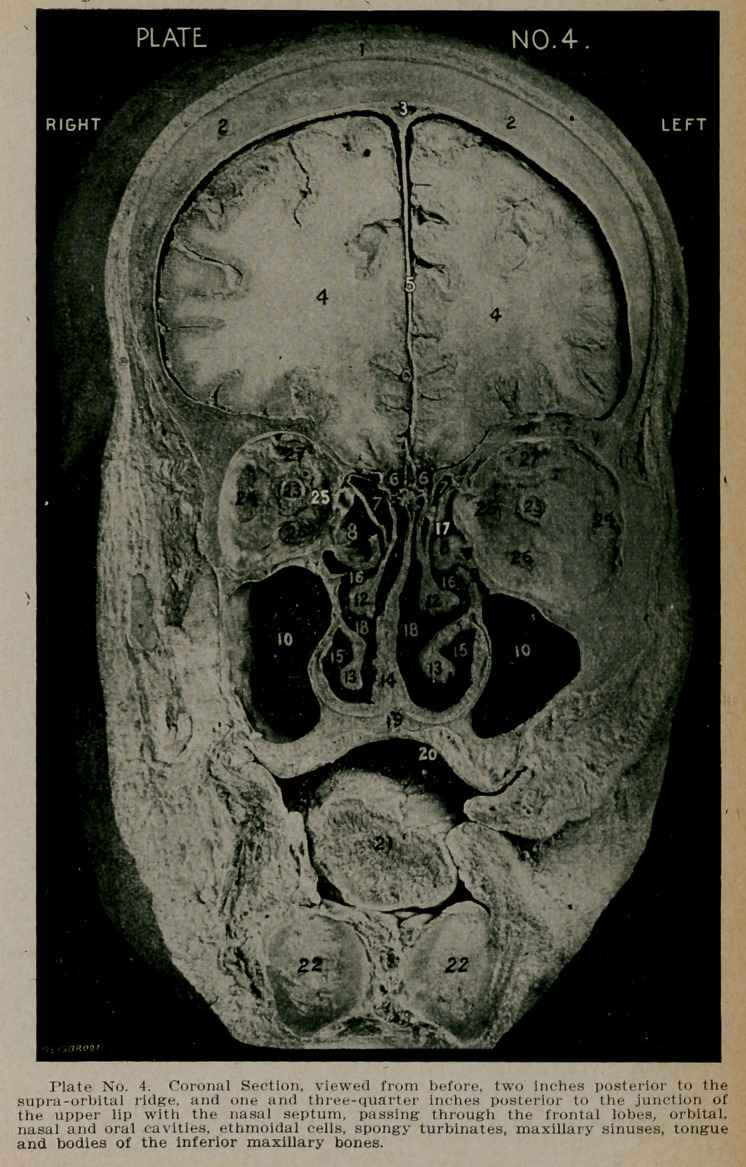


**Plate No. 5. f5:**
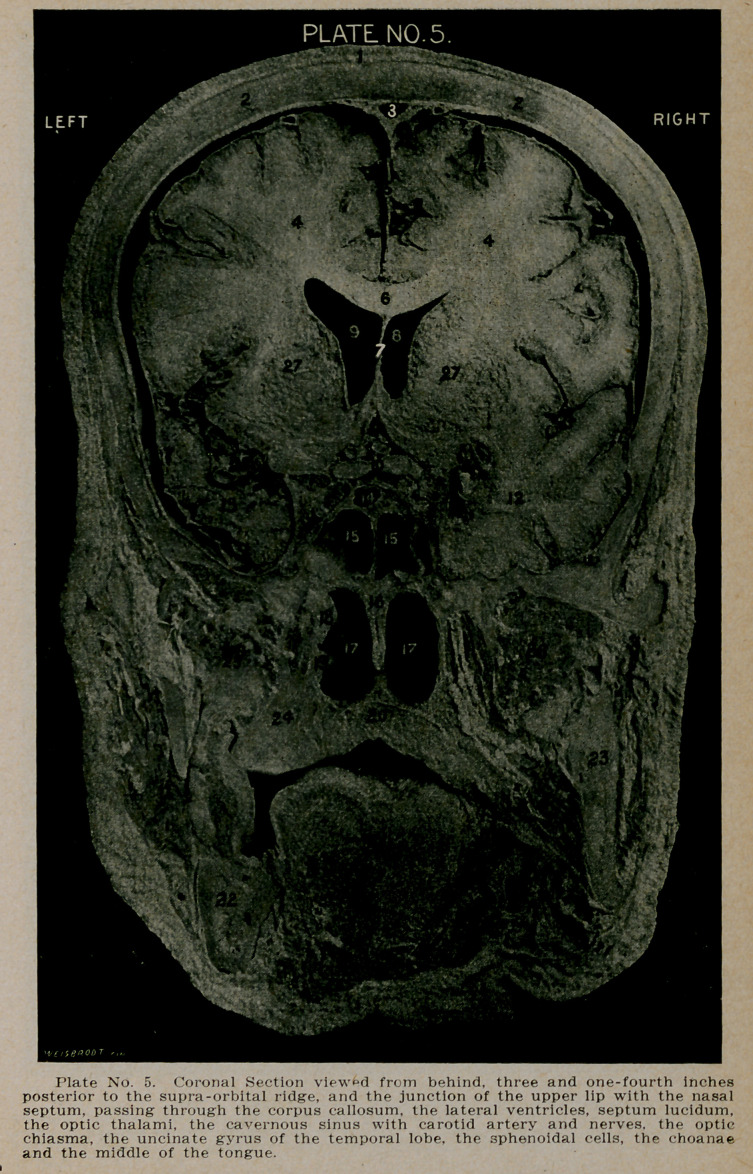


**Plate No. 6. f6:**